# Regulation of Recombination between *gtfB/gtfC* Genes in *Streptococcus mutans* by Recombinase A

**DOI:** 10.1155/2013/405075

**Published:** 2013-02-17

**Authors:** Satoko Inagaki, Kazuyo Fujita, Yukiko Takashima, Kayoko Nagayama, Arifah C. Ardin, Yuki Matsumi, Michiyo Matsumoto-Nakano

**Affiliations:** ^1^Department of Pediatric Dentistry, Osaka University Graduate School of Dentistry, 1-8 Yamada-oka, Suita, Osaka 565-0871, Japan; ^2^Department of Pediatric Dentistry, Okayama University Graduate School of Medicine, Dentistry and Pharmaceutical Sciences, 2-5-1 Shikata-cho, Kita-ku, Okayama 700-8558, Japan

## Abstract

*Streptococcus mutans* produces 3 types of glucosyltransferases (GTFs), whose cooperative action is essential for cellular adhesion. The recombinase A (RecA) protein is required for homologous recombination. In our previous study, we isolated several strains with a smooth colony morphology and low GTF activity, characteristics speculated to be derived from the GTF fusions. The purpose of the present study was to investigate the mechanism of those fusions. *S. mutans* strain MT8148 was grown in the presence of recombinant RecA (rRecA) protein, after which smooth colonies were isolated. The biological functions and sequences of the *gtfB* and *gtfC* genes of this as well as other clinical strains were determined. The sucrose-dependent adherence rates of those strains were reduced as compared to that of MT8148. Determination of the sequences of the *gtfB* and *gtfC* genes showed that an approximately 3500 bp region was deleted from the area between them. Furthermore, expression of the *recA* gene was elevated in those strains as compared to MT8148. These results suggest that RecA has an important role in fusions of *gtfB* and *gtfC* genes, leading to alteration of colony morphology and reduction in sucrose-dependent adhesion.

## 1. Introduction

*Streptococcus mutans* is known to be a primary causative agent of dental caries in humans [1]. One of the important virulence properties of the bacterium is its ability to form biofilm, known as dental plaque, on tooth surfaces [2]. *S. mutans* has been shown to produce 3 types of GTFs (GTFB, GTFC, and GTFD), whose cooperative action is essential for cellular adhesion [3]. Environmental conditions encountered by *S. mutans* in dental biofilms are highly variable, including frequent shifts in pH from above 7.0 to as low as 3.0 during the ingestion of dietary carbohydrates by the host [4]. Thus, pH exerts significant ecological pressure on *S. mutans*, and its ability to tolerate and grow in low pH environments is crucial for its survival and pathogenicity. 

 Recombinase A (RecA) is essential for transformation of both plasmid and chromosomal DNA in *Streptococcus pneumoniae* [5]. The *recA* gene is required for genetic transformation and is directly regulated by the cell signaling mechanism that induces competence in *S. pneumoniae* [6]. In addition, homologous recombination is strictly dependent upon the presence of the RecA protein [7]. However, RecA function remains uncharacterized due to the complexity of the recombination process. RecA-dependent recombination of the *gtfB* and *gtfC* genes has been reported to occur at a frequency of 10^−3^ in *S. mutans* [8], while these activities of GTFB and GTFC were found to be significantly reduced as compared to the reference strains [9]. On the other hand, it was also reported that spontaneous *gtfB*-*gtfC* recombination in *S. mutans* is not dependent on RecA and that a variety of *in vivo* generated *gtfB*-*gtfC* recombinants have similar sites of recombination [10]. However, biofilm formation by a RecA-deficient mutant strain was reduced as compared to that of the parental strain [11], and RecA was shown to have a relationship with expressions of the genes or proteins involved in the response to pH level by *S. mutans*, as well as expressions of GTFB and GTFC activities [11, 12]. In the present study, we demonstrated that the *recA* gene influences *gtf* expression, while the relationship between the *recA* gene and *gtfB*-*gtfC *recombination was shown using recombinant RecA protein and clinical isolates.

## 2. Materials and Methods

### 2.1. Bacteria Strains

The strains used in this study are listed in Table 1. All of the procedures in the present study were approved by the Ethical Committee of the Osaka University Graduate School of Dentistry. Each was grown in brain heart infusion (BHI) broth, Todd Hewitt (TH) broth, or Mitis-Salivarius (MS) agar (Becton-Dickinson, Franklin Lakes, NJ, USA).

### 2.2. Expression and Purification of Recombinant RecA (rRecA) Protein

rRecA was generated using a method previously described [13]. Briefly, *recA* fragments were amplified from genomic DNA of strain MT8148 by PCR using appropriate primers (recA/PF1: 5′-GGT GAT GAG CGT AAG AAA GC-3′, recA/PR1; 5′-TGG ATA ACC GCC TGC CCC AAG AGC-3′), then subcloned into the expression vector pET42a (+) (Novagen, Darmstadt, Germany). The plasmids were transformed into *Escherichia coli* BL21 (DE3) (Novagen). *E. coli* BL21 (DE3) organisms harboring the recombinant plasmids were grown in Luria-Bertani broth and protein expression was induced by adding isopropylthio-*β*-D-galactoside (Wako Pure Chemical Industries, Osaka, Japan). The cells were harvested by centrifugation, then pelleted cells were suspended in phosphate-buffered saline buffer (PBS; pH 7.4) and sonicated on ice. Recombinant RecA (rRecA) proteins were obtained by centrifugation. Supernatants were applied to a glutathione Sepharose 4B column (Amersham Pharmacia Biotech Inc., Piscataway, NJ, USA) and eluted with 10 mM of glutathione buffer (50 mM Tris-HCl, 10 mM glutathione, pH 8.0), followed by dialysis with Milli-Q water. Purified rRecA was stored in aliquots at −80°C.

### 2.3. Assay for Recombination Frequency

The test strains were grown in TH broth for 18 hours and inoculated into a 1/100 volume of fresh TH broth. rRecA protein (2 mg/mL) was added to the experimental group, then the strains were grown for 18 hours at 37°C in TH broth. Finally, they were inoculated onto MS agar plates and cultured anaerobically at 37°C for 48 hours to confirm culture purity and colony morphology. This experiment was independently repeated 3 times.

### 2.4. Expression of GTF

 Sodium dodecyl sulfate-polyacrylamide gel electrophoresis (SDS-PAGE) and western blot analyses were performed to determine the expression of the *gtf *gene in the tested strains, according to a method previously described by Aoki et al. [16]. The tested organisms were grown in BHI broth at 37°C to an OD550 value of 1.0. Cells were collected by centrifugation and the pellet was washed with PBS, then the bacterial cells were dissolved in SDS gel loading buffer. Next, an equal amount of each protein was separated by 7% SDS-PAGE and transferred onto a polyvinylidene difluoride membrane (Immobilon, Millipore, Billerica, MA, USA). GTFB and GTFC were detected using a rabbit anti-GTF antibody [17], as well as swine anti-rabbit immunoglobulin conjugated with alkaline phosphatase (Dako, Glostrup, Denmark).

### 2.5. Determination of GTF Activity

The enzyme activities of GTF protein were determined using polyacrylamide gels, as previously described [18]. Briefly, the strains were grown to the same OD550 value of 1.0 and cells were collected. Next, the cells were washed with PBS, then resuspended in phosphate-buffered saline buffer and adjusted to the same OD550 of 1.0. Fifteen microliters of each cell suspension was run on 7.5% SDS-PAGE gels. After electrophoresis, the gels were incubated overnight at 37°C in 3% sucrose, 0.5% Triton X-100, and 10 mg/mL dextran T10 in 10 mM sodium phosphate, pH 6.8, at 37°C, and the resulting glucan bands were treated with periodic acid and pararosaniline. The intensities of the stained bands were used to determine the activities of the GTF proteins. 

### 2.6. Sucrose-Dependent Adherence Assay

Sucrose-dependent adherence of *S. mutans* growing cells was determined using a turbidimetric method, as previously reported [14]. The test strains were grown in BHI broth containing 1% sucrose at 37°C for 18 hours at a 30° angle. After incubation, the culture tubes were vigorously vibrated with a vortex mixer for 3 seconds, and nonadhesive cells were transferred to fresh tubes. Cells remaining on the glass surface (adhesive cells) were removed by a rubber scraper and suspended in 3 mL of distilled water. Both adhesive and non-adhesive cells were dispersed by ultrasonication; then the turbidity of the suspension was determined by reading optical density at 550 nm. The cells were defined as OD550 (adhesive cell plus non-adhesive cells) and the percentage of adherence was defined as 100 × OD550 (adhesive cells)/OD550 (total cells). All assays were performed 3 times, with the mean and standard deviation presented. 

### 2.7. Specification of Location of *gtfBC* Recombination

PCR analyses were performed to identify the recombination of *gtfB*-*gtfC* gene fusions in all strains using appropriate primers (gtfB-LAF: 5′-CAG TTT AAA ATT TGG AGG TTC CTA ATG GAC-3′, gtfC-LAR: 5′-AAG AAG CCT GAG AAA TTT ACA GCT CAG ACT-3′). Cloning of this region was achieved by LA-PCR amplification of *gtfBC* gene fusions and ligation to a TOPO LA vector (Invitrogen, Carlsbad, CA, USA). Clones containing a full-length sequence were sequenced in both directions with universal M13 primer, as well as gtfB and gtfC primers (Table 2). Data analysis was performed using Gene Works software (IntelliGENETICS). The sequences of each strain were compared using multiple alignment analysis with CLUSTAL W from the DNA Data Bank of Japan (DDBJ, Mishima, Japan) [19]. 

### 2.8. Real-Time Quantitative RT-PCR

Primers for 16SrRNA were also designed as internal controls. Total RNA was isolated from mid-log-phase cell cultures (15 mL). After centrifugation, the cells were suspended in 0.3 mL of diethylpyrocarbonate-treated water. The samples were transferred to FastRNA tubes with blue caps (Qbiogene, Inc., Carlsbad, CA, USA); then 0.9 mL of TRIzol reagent (Invitrogen) was added. Cells were broken using a FastPrep FP120 homogenizer (Qbiogene) at a speed setting of 0.6 for 30 s. After the samples were placed on ice for 2 minutes, 0.2 mL of chloroform was added and the tubes were vortexed and centrifuged again, as described above. RNA was finally precipitated from the aqueous phase with isopropanol, and the resulting pellets were dried and resuspended in 20 *μ*L of diethylpyrocarbonate-treated water. For reverse transcription-PCR (RT-PCR) analysis, RNA samples were treated for 15 minutes at 37°C with 1.0 U/mL of RNase-free DNase (Amersham Biosciences) to remove contaminating DNA. Reverse transcription was carried out with SuperScript III (Invitrogen) according to the directions of the supplier. Real-time RT-PCR was performed using cDNA samples with either 16S rRNA or specific primers (recART-F; 5′-GGA TCC GAG AAA AAG ATT GGC CAA AAG AAT-3′, recART-R; 5′-TAA AGA CTC GGG CTT GGG ACC TAT TTT TAT-3′) using IQ-Supermix PCR reagent (Bio-Rad Laboratories, Hercules, CA, USA) in an iCycler thermal cycler according to the manufacturer's recommendations (Bio-Rad). Relative expression levels of the target gene transcripts were then calculated by normalizing the levels of specific RNA of each target gene with the levels of 16S rRNA. By normalizing the Ct values for the target genes to the total amount of 16S rRNA, all samples were compared and the relative fold changes in the samples were calculated using the −ΔΔCt method described for the MyIQ real-time PCR detection system (Bio-Rad).

### 2.9. Statistical Analysis

Intergroup differences of various factors were estimated by a statistical analysis of variance (ANOVA) for factorial models. Fisher's protected least-significant difference test was used to compare individual groups. Statistical computations were performed using STAT-VIEW II (Macintosh computer application).

## 3. Results

### 3.1. Frequency of Smooth Colony Morphology

**Figure 1:**
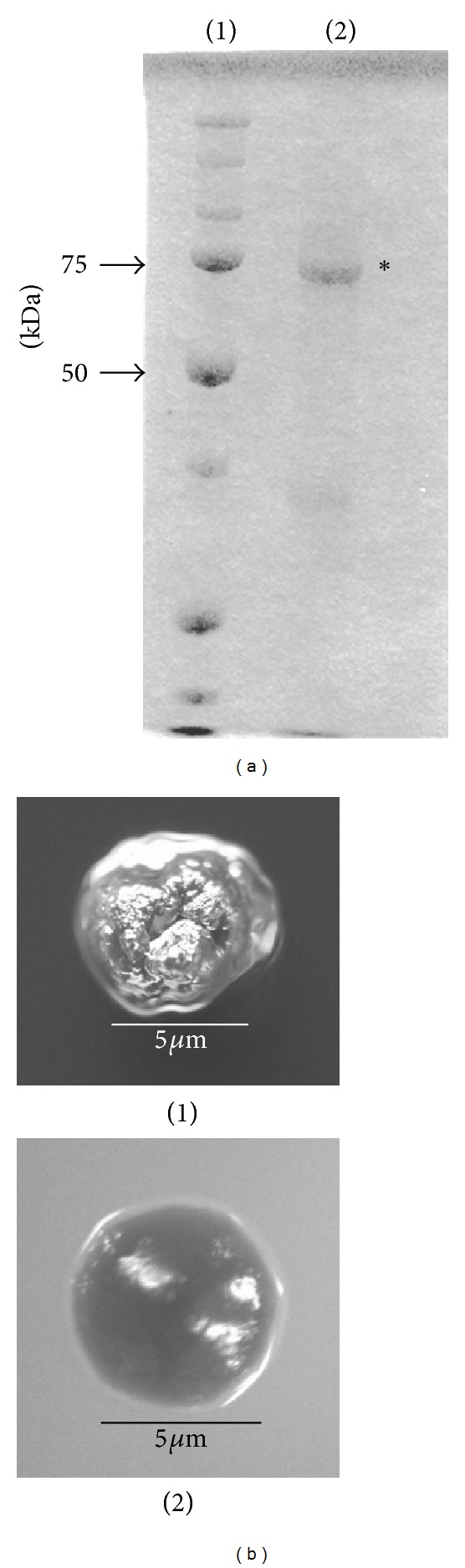
Coomassie blue staining of rRecA (a) and colony morphology of strains MT8148 and RRA1 (b). Lane 1: molecular marker, Lane 2; rRecA (asterisk). (b) (1) MT8148, (2) RRA1.

The purified rRecA protein with a molecular weight of approximately 70 kDa is shown in Figure 1(a). Typical rough colonies were observed on MS-agar plates with no addition of rRecA in the test tubes after inoculation of the overnight grown culture of MT8148 was performed (Figure 1(b)). In contrast, the addition of rRecA to the test tubes after inoculation of MT8148 resulted in smooth colonies at a frequency of 0.2% (Figure 1(b)). 

### 3.2. GTF Expression

**Figure 2:**
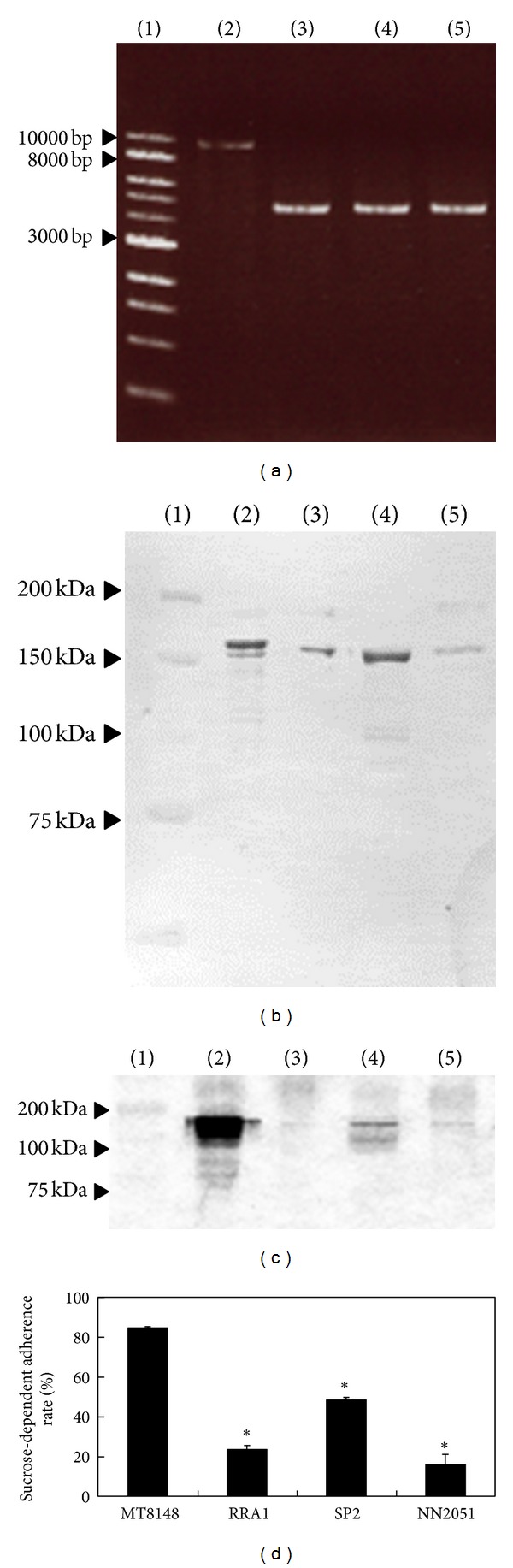
PCR amplification of *gtfB* and *gtfC* regions (a), western blot analysis (b), activity staining (c), and sucrose-dependent adherence (d) of MT8148, RRA1, and 2 clinical strains. Lane 1: molecular marker, Lane 2; MT8148, Lane 3; RRA1, Lane 4; SP2, Lane 5; NN2051. There were significant differences between MT8148 and the other strains (*P < 0.001).

PCR analysis using primers revealed the *gtfB*-*gtfC* region of RRA1, a mutant isolated from MT8148 grown overnight with rRecA added, and clinical strains SP2 and NN2051, which possessed *gtfBC* recombination and developed smooth colonies. The molecular size of that in these strains was approximately 4500 bp, significantly less as compared to that in MT8148 (approximately 9000 bp) (Figure 2(a)). In addition, western blot analysis of cell-associated GTFs of these strains with a smooth appearance revealed only a single band between the position of corresponding GTFB and GTFC (Figure 2(b)). Activity staining demonstrated that the levels of GTF activities of these strains with a smooth appearance were lower than that of MT8148 (Figure 2(c)). Furthermore, the rates of adherence of these strains to glass in the presence of sucrose were significantly lower than that of MT8148 (Figure 2(d)). 

### 3.3. Sequence Analysis of *gtfB-gtfC* Region

**Figure 3:**
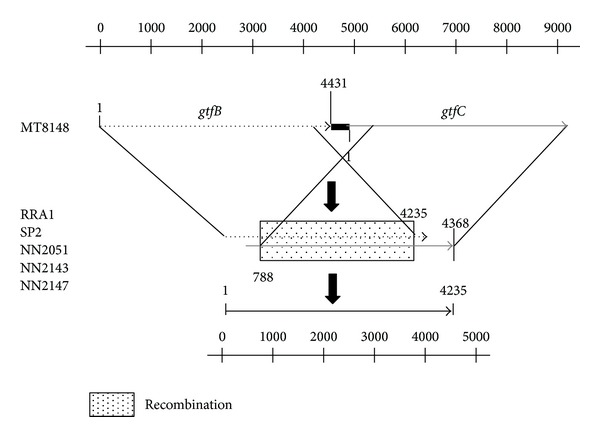
Illustration of *gtfB*-*gtfC* gene location in MT8148 and fusion strains. The recombination was found located at a position 4235 bp downstream of *gtfB* and 788 bp upstream of *gtfC*. The *gtfB* and *gtfC* genes used are listed in DDBJ as accession No. D88651 and D88652.

Figure 3 shows the regions of recombination of *gtfB* and *gtfC* in RRA1, SP2, NN2051, NN2143, and NN2147. Sequence analyses revealed that the total length of the *gtfB*/*gtfC* region in these strains was 4368 bp. In addition, the nucleotides of *gtfB* at the 4235 bp position and those of *gtfC *at 788 bp were found to be deleted. The area of recombination of *gtfB*/*gtfC *is shown in Figure 3. 

### 3.4. Expression of *recA* Gene

**Figure 4:**
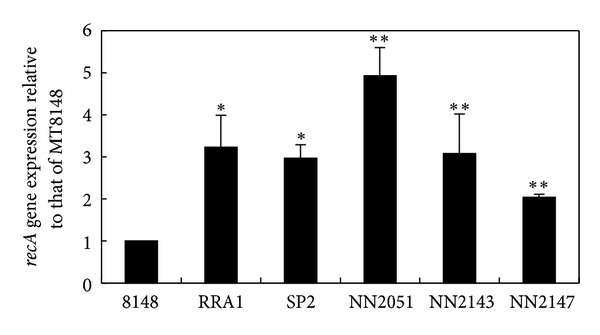
*recA* expression in all strains was examined by RT-PCR. There were statistically significant differences between MT8148 and the other strains (*P < 0.05, **P < 0.001, Fisher's PLSD).

Quantitative RT-PCR analyses revealed that the *recA* expression was significantly higher in RRA1, SP2, NN2051, NN2143, and NN2147 as compared to MT8148 (Figure 4). 

## 4. Discussion

 In our previous study, the *recA* gene was shown to have a relationship with the activities of GTF and expression of genes encoding GTF [11]. It has been reported that homologous recombination is strictly dependent on the presence of the RecA protein [7]. In the present study, the addition of rRecA protein increased the frequency of spontaneous recombination of *gtfB* and *gtfC*. Furthermore, the morphological appearance of those strains was altered to smooth and their GTF activities were reduced. We also confirmed the location of the recombination of *gtfB* and *gtfC* by sequence analysis. It is of great interest that the recombination region of RRA1 and those of the clinical strains with smooth colony morphology, such as SP2 and NN2051, were the same. These results suggest that recombination of *gtfB *and *gtfC* genes is associated with RecA, and that excess gene expression may cause fusions between *gtfB* and *gtfC*.

 GTFs of *S. mutans* are cell surface proteins that facilitate adherence and colonization. Although the roles of GTFB and GTFC in *S. mutans* for virulence related to dental caries are well established, the mechanisms that control expression of these proteins are poorly understood. The *gtfB* and *gtfC* genes appear in tandem sequence, whereas the promoter regions are upstream of the GTFB and GTFC open reading frames. In our previous studies, we found several clinical strains that developed smooth colonies with low GTF activities [9]. Morphological changes of mutant colonies observed after culturing on sucrose-containing agar plates suggest alterations in the production of extracellular polysaccharides or cell surface proteins. In addition, we examined the expressions of genes that encode various surface proteins such as glucan-binding proteins of *S. mutans* and observed changes in *gtfB* and *gtfC* expressions in the clinical strains as compared to the reference strain MT8148. 

 In the present study, we revealed expressions of the *recA* gene and other stress response genes in clinical strains using real-time RT-PCR. Interestingly, *recA* gene expression was elevated in all of the clinical strains tested, while the levels of the other stress response genes such as the *dnaK* [20] and *groEL* [21] genes were unchanged in those strains (data not shown). Hazlett et al. [10] reported that inactivation of the *gbpA* gene encoding the glucan-binding protein of *S. mutans* promoted *in vivo* recombination of *gtfB* and *gtfC*, and that this recombination was independent of the *recA* gene, while *gtfBC* recombinants had similar sites of recombination. Although they did not determine the mechanism for accumulation of *gbpA* mutants with *gtfB*-*gtfC* fusion, the cell surface structure seems to be important for maintaining a steady state of intact *gtfB* and *gtfC* genes. However, the *recA* gene is one of the stress response genes involved in quorum sensing in *S. pneumoniae* [6]. Thus, inactivation of *gbpA* may influence the expression of genes associated with quorum sensing relative, including *recA*. Furthermore, *gtfB *and *gtfC* fusion was also found in *rml* mutant strains, in which inactivation of the *rml* gene caused the fusion [22]. However, the present clinical strains with smooth colonies also demonstrated *rml* gene expression (data not shown). Taken together, *gtfB* and *gtfC* fusion may occur by some alterations of gene expression in *S. mutans*. 

 We speculated that fusion between *gtfB* and *gtfC* may occur under an acidic condition, since *recA* gene expression was shown to be elevated at acidic pH levels [11]. It was also reported that excess *recA* gene expression related to low pH is caused by drastically changed pH in the human oral cavity. Such a condition leads to increased *gtfB*-*gtfC* fusions by binding RecA to the junction of the *gtfB* and *gtfC* genes. *S. mutans *utilizes quorum-sensing systems to modulate environmental stress responses, such as low pH, antibiotics, and oxidative stress [23]. Therefore, we speculated that DNA recombination and phenotypic change through the uptake of extracellular RecA may have a relationship with the signal transduction system. Additional studies are required to clarify this point.

## Figures and Tables

**Table 1:** Strains used in this study.

Strain	Sero type	Colony morphology	Characteristic	Reference
MT8148	*c *	Rough	Oral isolate from Japanese child	[14]
RRA1	*c *	Smooth	Mutant of MT8148 isolated in presence of rRecA in culture medium	This study
SP2	*c *	Smooth	GTFBC fusion strain, *S. mutans* colonization- defective mutant recombination between *gtfB *and *gtfC* genes	[8]
NN2051	*c *	Smooth	GTFBC fusion strain, isolated from 10-year-old girl	[15]
NN2143	*e *	smooth	GTFBC fusion strain, isolated from 15-year-old girl	[15]
NN2147	*c *	smooth	GTFBC fusion strain, isolated from 10-year-old girl	[15]

**Table 2:** Primers used for determination of nucleotide alignment of *gtfB*-*gtfC* region.

Primer	Sequence (5′-3′)
gtfB-LAF	CAG TTT AAA ATT TGG AGG TTC CTA ATG GAC
LA1/R	ATT GGC TGC ATT GCT ATC ATC
LA1/F	CAA CCG AAG CTG ATA CAG ATG
LA2/R	CAG CTG TCA AAT AAT GAT CAA CAT G
LA2/F	TGG TAT CGT CCT AAG TAC ATC TTG
LA3/R	GAT ACG GTA GTT GGA ATT TGC
LA3/F	GCT AAT TCC AAC TAC CGT ATC
LA4/R	GAG GAT TCA TGC CTG AAC GTT G
LA4/F	CAA CGT TCA GGC ATG AAT CCT C
LA5/R	TTA AGC AGG GTT TCG ATG GCT TCG
LA5/F	CGA AGC CAT CGA AAC CCT GCT TAA
LA6/R	CAG CGG CAG CGC CTA CTG GAA CCC
LA6/F	GGG TTC CAG TAG GCG CTG CCG CTG
LA7/R	TCA GGC ACC CAG TCA GCC ATT ACC
LA7/F	CGG GAC AGC CGA TGA TTT GGT G
LA8/R	GTT CCG TGA TTT GGG TTA ATC AAC G
LA8/F	CGT TAG TTA ACC CAA ATC ACG GAA C
LA9/R	GCA CCA TGA ACA CGT GTA TTG CCG AC
LA9/F	CAA CTG CTG ATG GAA AGC TGC G
LA10/R	CTC TCC CTT AGC CTG AAC ACC
LA10/F	GGT GTT CAG GCT AAG GGA GAG
gtfC-LAR	AAG AAG CCT GAG AAA TTT ACA GCT CAG ACT
